# Malignant Tumours Mimicking Complicated Appendicitis and Discovered upon Follow-Up after Percutaneous Drainage: A Case of Two Patients

**DOI:** 10.1155/2017/3253928

**Published:** 2017-11-16

**Authors:** Sharandran Chandra Mohan, Krishna Mohan Gummalla, Martin Weng Chin H'ng

**Affiliations:** Diagnostic Radiology Department, Tan Tock Seng Hospital, 11 Jalan Tan Tock Seng, Singapore 308433

## Abstract

The conservative management of periappendiceal abscesses is gaining favour due to decreased morbidity and improved clinical outcomes for patients. Occasionally however an abscess can mask underlying sinister pathology. In this article, we highlight two cases of appendiceal adenocarcinoma that were initially diagnosed as periappendiceal abscesses and managed conservatively with percutaneous drainage. We also discuss clinical and imaging features that may assist with identifying a hidden malignancy when presented in these situations.

## 1. Introduction

Patients presenting with periappendiceal abscesses are generally treated conservatively [1]. A drain is inserted under radiological guidance until the abscess resolves. Periappendiceal abscesses are usually due to acute appendicitis. However, appendiceal malignancy may also present with periappendiceal abscesses and it becomes difficult to differentiate between these two. Periappendiceal abscess due to malignancy can be recurrent or persistent despite drain insertion. Follow-up is advised after drain insertion, as once the abscess resolves there may be unmasking of an underlying malignancy.

Approximately 20 patients underwent image guided drainage in our institution for periappendiceal collections between 2013 and 2015 (2 years). We present two cases where the initial diagnosis was periappendiceal abscess due to acute appendicitis and a drain was inserted. There was persistent abscess collection despite drainage in these cases and subsequent histology revealed an underlying malignancy.

## 2. Case Report

### 2.1. Case  1

**Figure 1:**
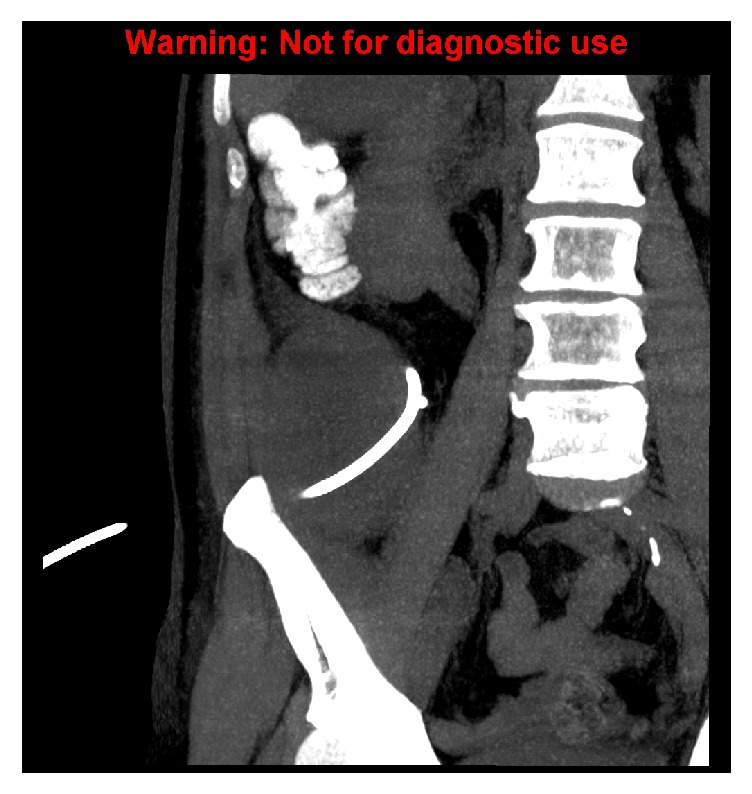
Coronal reconstruction demonstrating a drainage tube within the appendiceal collection.

A 76-year-old man presented with a one-week history of intermittent fever, nausea, and right iliac fossa (RIF) pain. Initial CT scan performed at an external centre showed a large irregular collection in the RIF, compatible with an abscess, likely from a perforated appendix. He was referred to our institution and underwent CT-guided drainage of the collection (Figure 1). The amount of drainage gradually resolved and the tube was removed after 18 days.

**Figure 2:**
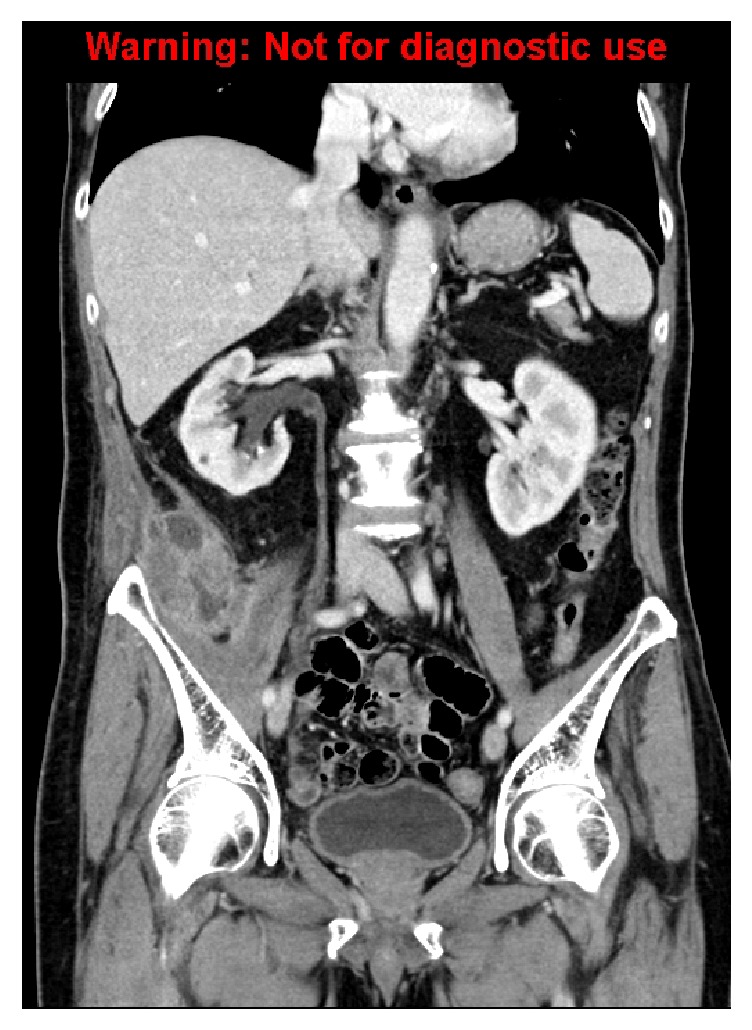
Coronal reconstruction demonstrating a persistent abscess in the retrocaecal region, with new extension to involve the pelvic side wall and iliopsoas muscles.

An outpatient CT scan performed one month after the drainage for recurrent symptoms revealed a persistent abscess in the retrocaecal region, now extending to involve the pelvic side wall and iliopsoas muscles (Figure 2). The patient was managed with a repeat drainage of the primary collection.

Another follow-up in the general surgery clinic showed persistent symptoms and continuous drain output, and he underwent an extended right hemicolectomy approximately 3 months from the date of initial presentation. Intraoperative findings revealed a large, polypoid caecal tumour with a 2 cm defect and contained abscess posteriorly extending into the lateral abdominal wall, close to the iliac crest. Histology confirmed this to be a mucinous adenocarcinoma.

### 2.2. Case  2

**Figure 3:**
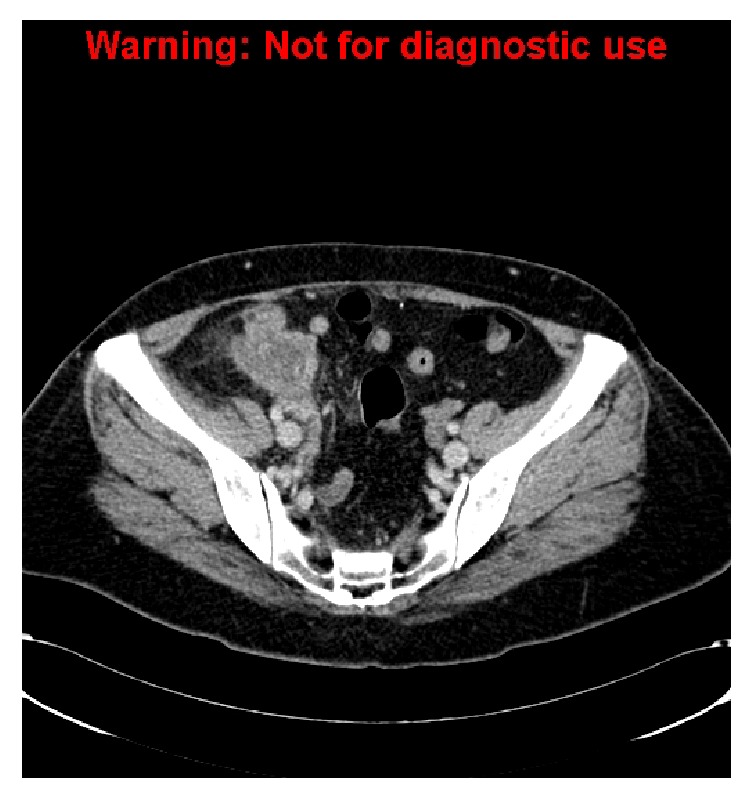
Thickened and inflamed appendix with a focal perforation of the tip with an adjacent abscess.

A 49-year-old lady presented to our emergency department with a one-week history of fever and localised RIF pain. CT scan performed on admission delineated a dilated and ill-defined appendix with focal perforation at its tip (Figure 3). There was an adjacent abscess with surrounding fat-stranding as well as reactive thickening of the caecum and terminal ileum with prominent ileocolic lymph nodes. The patient was initially treated conservatively with intravenous antibiotics.

**Figure 4:**
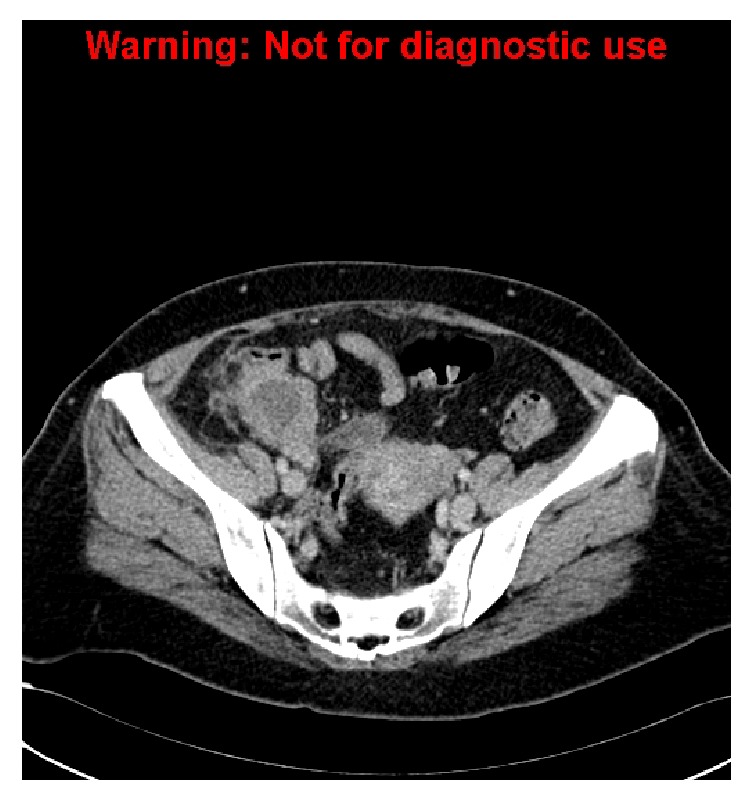
Repeat CT scan demonstrates a largely stable periappendiceal collection.

She represented approximately 2 weeks later due to worsening symptoms. Repeat CT scan (Figure 4) demonstrated a stable collection and CT-guided drainage was performed. As the drain output progressively decreased, this tube was removed after 7 days.

**Figure 5:**
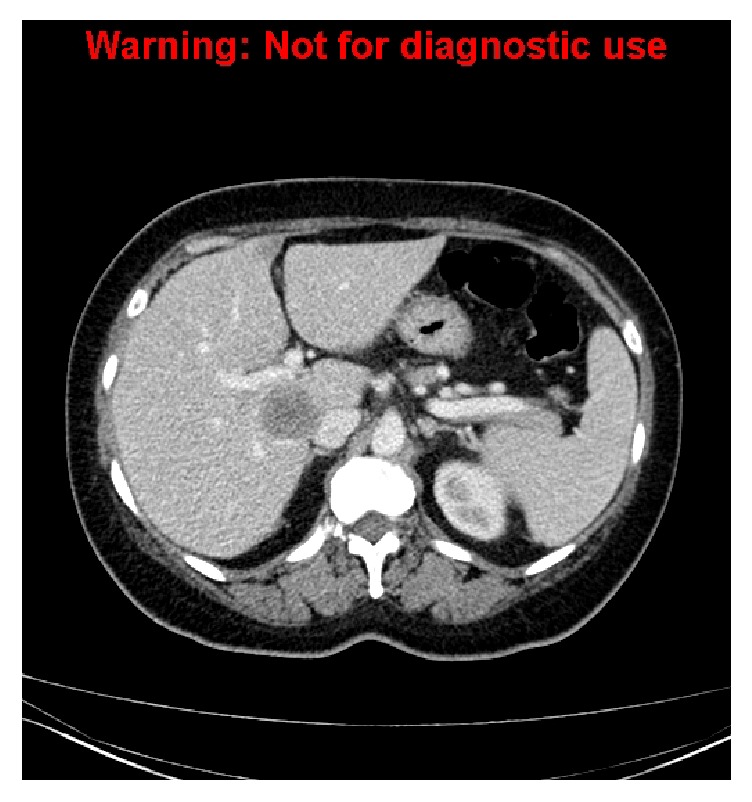
A new hypodense lesion in caudate lobe of the liver suspicious for metastasis.

**Figure 6:**
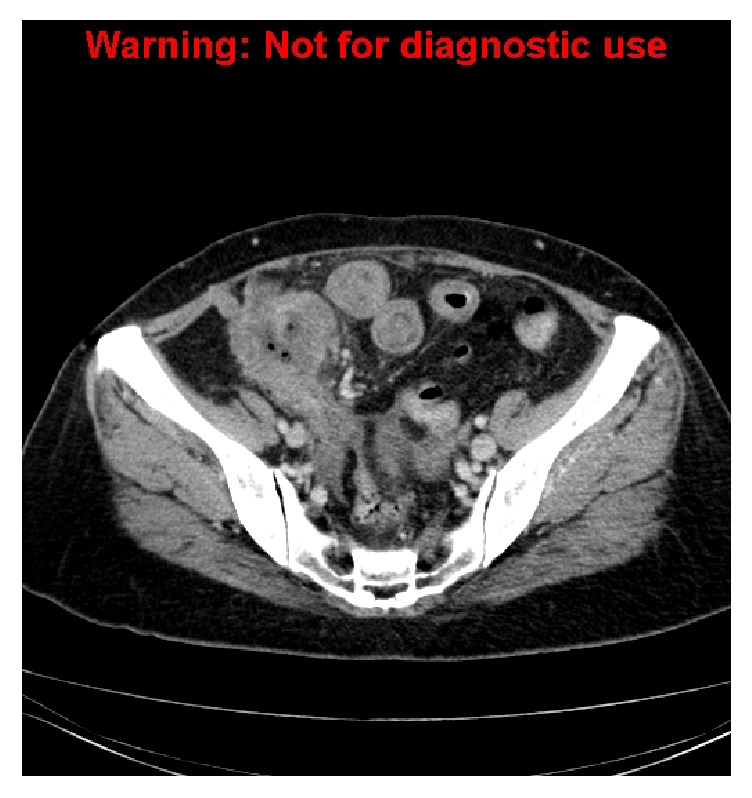
A persistent periappendiceal collection is again seen. New findings include small bowel obstruction as well as nodules adjacent to the collection and along the track of previous drain insertion.

She returned another two and a half months later for nonresolving symptoms. The repeat CT scan (Figure 5) revealed an irregular lobulated mass with central necrosis at the ileocaecal junction associated with small bowel obstruction. New small soft-tissue nodules were observed adjacent to the mass and along the track of previous drain insertion. The adjacent mesenteric lymph nodes were enlarged and necrotic. A new hypodense lesion in the caudate lobe of the liver was also identified, suspicious for a metastasis (Figure 6).

The patient underwent an open right hemicolectomy with en bloc right salpingooophorectomy. Intraoperative findings revealed a mass involving and encasing the terminal ileum as well as involving the right fallopian tube, ovary, and ureter and extending into the pelvic side wall. A separate appendix was not identified within the mass. Multiple peritoneal nodules were evident in the omentum and as far up as the hepatic dome. The histology specimen was confirmed to be an adenocarcinoma.

## 3. Discussion

Acute appendicitis is the result of luminal obstruction. This is typically caused by faecolith or lymphoid hyperplasia and less frequently by foreign body impaction or parasites [2]. A small subset of acute appendicitis is caused by primary appendiceal malignancies such as carcinoid tumours, adenocarcinoma, Kaposi sarcoma, and lymphoma and less frequently due to metastases from breast or colonic tumours [2, 3]. Perforation with abscess formation is an infrequent but important complication of appendicitis as it leads to increased morbidity and mortality [4].

CT scans are routinely performed nowadays both to diagnose acute appendicitis and to identify mimics such as right ureteric calculus, epiploic appendagitis, torsion of a Meckel's diverticulum, mesenteric adenitis, inflammatory bowel disease, colitis, gynaecological disorders, and right-sided diverticulitis [4].

Current literature supports nonsurgical management of appendiceal abscesses [1, 4, 5]. This reduces morbidity and mortality. Antibiotic therapy is the mainstay of treatment and percutaneous drainage is performed in cases where there is a large or nonresolving collection. In cases of periappendiceal abscess due to underlying malignancy, there can be recurrent or persistent collections despite drainage [3]. A decrease in the size of the collection may unmask the underlying mass lesion.

A case report by Fusari et al. described findings of acute appendicitis on a preoperative CT scan with a loculated fluid collection and lymphadenopathy adjacent to the appendix that was found to be a signet cell carcinoma on histology [6]. Jongsma and Puylaert presented a case in which a lesion initially thought to be an appendiceal abscess was later found to be a complicated appendiceal mucocoele [7]. Another case report by Fiume et al. describes a caecal adenocarcinoma presenting as an appendiceal abscess [8]. Our cases similarly illustrate two examples of appendiceal malignancy presenting as appendiceal abscesses which were both proven to be adenocarcinomas on histology.

Multiple case studies are available describing imaging findings of pathology at or near the appendix such as mucocoeles, mucinous epithelial neoplasms, soft-tissue masses with nonmucinous or colonic-type epithelial neoplasms, carcinoid tumours, and lymphoma causing diffuse mural thickening and dilation of the appendiceal lumen [9, 10]. These conditions may also result in abscesses, which are difficult to distinguish from those caused by perforated acute appendicitis. In patients with Crohn's disease, transmural inflammation and penetration of the bowel may also lead to abscess formation in the region of the terminal ileum [11].

Imaging alone however is inadequate for the follow-up of complicated appendicitis. There is evidence to support an early colonoscopy and an interval appendectomy in appropriate patients. A study by Lai et al. [12] showed that patients who had colon cancer associated with appendicitis had a higher stage and a greater incidence of distant metastasis. He recommended that patients older than 40 years should undergo an early colonoscopy to exclude the possibility of a coexistent colorectal cancer. Wright et al. [13] showed that appendiceal neoplasms were more frequent in patients undergoing an interval appendectomy after initial nonoperative management. This was most prevalent is patients above the age of 40, in whom 16% were found to have an underlying malignancy. They recommended an interval appendectomy as part of routine care and this view has been supported by other authors who have shared data showing that an interval appendectomy is a safe procedure [14].
